# Clinical Classification of Radiation Maculopathy as a Predictor of Functional Response to Intravitreal Dexamethasone Implant

**DOI:** 10.3390/jcm15187331

**Published:** 2026-09-21

**Authors:** Raffaele Parrozzani, Samuele Gava, Carolina Molin, Edoardo Midena, Giulia Midena

**Affiliations:** 1 Department of Neuroscience-Ophthalmology, University of Padova, 35128 Padova, Italy; raffaele.parrozzani@unipd.it (R.P.); samuele.gava@studenti.unipd.it (S.G.); carolina.molin@studenti.unipd.it (C.M.); 2 IRCCS-Fondazione Bietti, 00198 Rome, Italy; giulia.midena@fondazionebietti.it

**Keywords:** brachytherapy, macular edema, optical coherence tomography, radiation maculopathy, uveal melanoma

## Abstract

**Background**: Radiation maculopathy (RM) is a common complication after radiotherapy for intraocular tumors, causing permanent visual loss. Intravitreal dexamethasone (IV DEX) is effective for macular edema (ME) resolution, but functional outcomes remain highly heterogeneous. This study aimed to assess the prognostic utility of CEA classification on visual outcomes following IV DEX based on three parameters: largest cyst diameter (C), ellipsoid zone (EZ) disruption (E) and retinal pigment epithelium (RPE) atrophy (A). **Methods**: A retrospective analysis of 50 patients with RM secondary to Iodine-125 brachytherapy treated with IV DEX was performed. Best-corrected visual acuity (BCVA) and CEA parameters were analyzed with OCT before and after treatment. **Results**: IV DEX induced significant anatomical improvement (mean Δcyst: −203.3 μm, *p* < 0.001), regardless of functional response. Visual outcomes were related to baseline CEA stratification: eyes with intact outer retina gained +6.4 ETDRS letters (*p* = 0.002); eyes with EZ disruption demonstrated stability (−2.8 letters; *p* = 0.183); eyes with RPE atrophy significantly worsened (−11.7 letters; *p* = 0.014). Baseline RPE atrophy was the strongest negative predictor (*p* = 0.007). Subgroup analysis of patients with intact outer retina identified a baseline BCVA cutpoint of ≤70 letters to predict clinically significant visual improvement (≥5 letters), demonstrating a functional ceiling effect. **Conclusions**: The CEA classification effectively stratifies functional response in RM. EZ disruption and RPE atrophy identify eyes unlikely to achieve visual improvement despite a reduction in ME, aiding clinical decision-making and establishing functional expectations while promoting a fundamental shift from an edema-driven management to a biomarker-centered approach.

## 1. Introduction

Uveal melanoma (UM) is the most common primary intraocular malignancy in adults, and radiation therapy currently represents the gold standard for its management [1,2,3,4]. Although local tumor control is achieved in approximately 91% of cases, treatment is frequently associated with significant ocular side effects [5,6,7,8]. Among these, radiation maculopathy (RM) is the leading cause of permanent visual loss, affecting 25% to 50% of patients within 2 to 5 years following treatment [7,8].

RM has traditionally been attributed to radiation-induced vascular endothelial damage and disruption of the blood–retina barrier. However, recent evidence has highlighted a pivotal role of neuroinflammation in the development of progressive radiation-related macular edema (RME) and photoreceptor damage [9,10]. In this setting, the intravitreal 0.7 mg dexamethasone implant (IV DEX) has emerged as an effective therapeutic option for RME, including cases refractory to anti–vascular endothelial growth factor (anti-VEGF) therapy [11,12]. Nevertheless, despite its proven ability to reduce RME and exudation, both clinical experience and published data consistently demonstrate a functional–anatomical dissociation, with highly variable visual outcomes despite morphological improvement [13,14]. To better explain this heterogeneity, optical coherence tomography (OCT) has become essential for the assessment of microstructural retinal changes in RM beyond RME quantification [15].

This approach recently led our group to propose an OCT-based clinical classification, originally termed the CJA classification and subsequently renamed the CEA classification, reflecting the current nomenclature of the inner segment/outer segment junction (J) as the ellipsoid zone (EZ) [16]. This grading system identified three key determinants of visual prognosis within the central 1 mm foveal area: the vertical size of the largest macular cyst (C), EZ disruption (E), and retinal pigment epithelium (RPE) atrophy (A).

The aim of the present study was to validate the CEA classification and to evaluate the association of each individual parameter with the overall functional outcome after IV DEX in a cohort of patients who developed RM following plaque brachytherapy for UM. Through this validation, we sought to definitively clarify the prognostic role of outer retinal integrity in determining visual outcomes.

## 2. Materials and Methods

### 2.1. Study Population

This retrospective, longitudinal, single-center cohort study was conducted in accordance with the tenets of the Declaration of Helsinki and was approved by the Institutional Review Board (N. 32n/AO/20 Prot. N. 63589-20). Written informed consent was obtained from all participants. Medical records of patients diagnosed with UM and treated with Iodine-125 plaque brachytherapy (total prescribed dose of 85 Gy to the tumor apex) who subsequently developed RM were reviewed. Patients were managed at the Ocular Oncology Center of the University of Padova between October 2020 and November 2025. The included patients represent a distinct, independent cohort with no overlap with the study population detailed in our previously published work [16]. The inclusion criteria were as follows: diagnosis of RM confirmed by OCT; treatment with at least one intravitreal 0.7 mg IV DEX for RM; availability of best-corrected visual acuity (BCVA) measurements; and high-quality OCT scans. According to our standard clinical protocol for monitoring response to IV DEX, all patients underwent a comprehensive ophthalmic evaluation at baseline and at 4 months (±2 weeks) after treatment. Only paired pre- and post-injection observations with complete data at both timepoints were included in the analysis. Exclusion criteria included the following: the presence of coexisting macular diseases; IV DEX administered for serous retinal detachment secondary to UM; significant media opacities; missing BCVA data; cataract or vitreoretinal surgery during the study period; previous ocular irradiation. Patients with tumors involving the macular region were also excluded.

### 2.2. OCT Assessment

**Figure 1:**
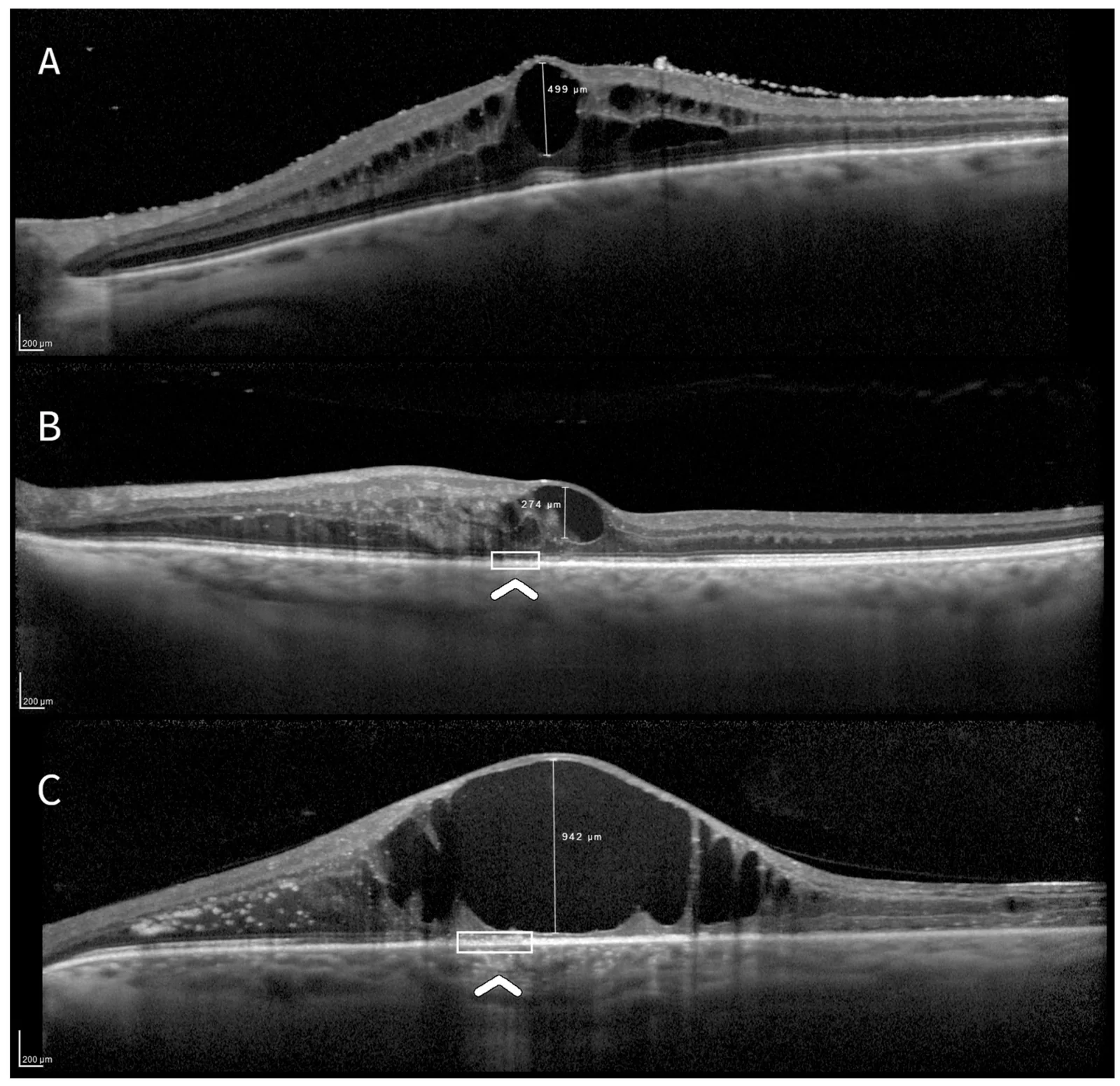
Representative OCT images illustrating the CEA classification in radiation maculopathy. (**A**) Group 1: radiation-related macular edema (RME) with a vertical diameter of the largest cyst of 499 µm, with preserved ellipsoid zone (EZ) and retinal pigment epithelium (RPE) integrity (C_499_E_0_A_0_); (**B**) Group 2: RME with a vertical diameter of the largest cyst of 274 µm, associated with ellipsoid zone disruption involving the foveal area (white arrow and rectangle) and preserved RPE (C_274_E_1_A_0_); (**C**) Group 3: severe RME with a vertical diameter of the largest cyst of 942 µm, extensive EZ disruption, and diffuse RPE atrophy (white arrow and rectangle) (C_942_E_1_A_1_).

All patients underwent OCT imaging using the Spectralis HRA+OCT system (Heidelberg Engineering, Heidelberg, Germany). For each injection event, a single high-resolution (HR) linear scan passing through the foveal center was analyzed. Scans were acquired with automatic real-time (ART) tracking ≥ 90 frames and a minimum image quality score of 30. Macular morphology within the central 1 mm foveal area was graded according to the previously published CJA classification [16], renamed the CEA classification to reflect the current nomenclature of the inner segment/outer segment junction as the EZ. The CEA classification is based on three OCT-derived parameters: the “C” component quantified the vertical diameter (µm) of the largest intraretinal cyst; the “E” component assessed EZ integrity, defined as the hyperreflective band corresponding to the junction between the photoreceptor inner and outer segments, and was categorized as intact (E0) or disrupted (E1); the “A” component evaluated the presence of RPE atrophy, defined by loss of the hyperreflective RPE band with increased signal transmission into the choroid, and was classified as absent (A0) or present (A1). To ensure grading reliability, CEA parameters were independently evaluated by two masked, experienced examiners (R.P. and S.G.). Discrepancies were resolved by consensus. Based on the combination of CEA parameters, each injection episode was stratified into three groups: group 1, preserved outer retinal integrity (no EZ disruption and no RPE atrophy); group 2, EZ disruption with preserved RPE; and group 3, combined EZ disruption and RPE atrophy. Because RM is a progressive disease, eyes were evaluated before each treatment and could contribute observations to multiple CEA groups across different injection events. BCVA was assessed before each OCT examination using Early Treatment Diabetic Retinopathy Study (ETDRS) charts and recorded as letter scores. An illustration of the OCT-based assessment of the CEA parameters is provided in Figure 1.

### 2.3. Statistical Analysis

For descriptive analyses of the overall cohort and subgroup comparisons, data are presented as means ± standard deviations. Interobserver agreement for categorical OCT parameters was assessed using Cohen’s Kappa. To account for repeated measures and multiple IV DEX injections per patient, variations in anatomical and functional parameters before and after treatment were analyzed utilizing linear mixed-effects models (LMMs). Specifically, the change in ETDRS letter score was used as the dependent variable, with baseline BCVA, EZ integrity, and RPE atrophy included as fixed effects, and patient identity modeled as a random intercept. In this case the significance was evaluated using Wald z-test. Furthermore, continuous variables were compared across groups utilizing ordinary least squares (OLS) regression models with cluster-robust standard errors clustered by patient. The assumptions of normality of the model residuals were verified through visual inspection of quantile-quantile (Q-Q) plots and residual-versus-fitted plots. Receiver operating characteristic (ROC) curve analysis using a mixed-effects logistic regression model with patient identity set as a random effect was performed to establish the optimal baseline BCVA cutoff predictive of a clinically significant visual gain (≥5 ETDRS letters) in group 1. The optimal threshold was determined using Youden’s J statistic. All statistical tests were two-sided, and a *p*-value < 0.05 was considered statistically significant. Where multiple comparisons were performed between clinical groups, a Bonferroni correction was applied. Statistical analyses were performed using Python (version 3.12; Python Software Foundation, Wilmington, Delaware, USA) with the SciPy (version 1.17.0) and Statsmodels (version 0.14.6) libraries.

## 3. Results

### 3.1. Population Features and Tumor Characteristics

A total of 92 IV DEX injection events in 50 eyes from 50 patients were included in the final analysis. In total, 29 (58%) patients were male and 21 (42%) were female, with a mean age of 61.2 ± 14.8 years (range 26.5–90.8). The mean number of injections per patient was 1.8 (range 1–6). According to the CEA classification, preservation of outer retinal integrity (group 1) was observed in 32 injection events (34.8%). The remaining 65.2% showed structural disruption of the outer retina, with isolated EZ disruption (group 2) in 37 events (40.2%) and combined EZ disruption and RPE atrophy (group 3) in 23 events (25.0%). Interobserver agreement was excellent for both categorical variables, with a Cohen’s Kappa value of 0.881 and 0.884 respectively for EZ integrity and RPE atrophy. In the overall cohort, mean baseline vertical cyst diameter was 355.3 ± 143.8 µm (range 0–942) and mean baseline BCVA was 57.3 ± 20.6 ETDRS letters (range 0–85). Mean time from treatment to RM presentation was 1.2 ± 0.4 years. Tumor and treatment characteristics are described in Table 1. The distribution of the number of injections per patient and the quantity of patients that contributed to a singular CEA group without transitioning between groups over time are summarized in Table 2. No statistically significant differences were found when evaluating each parameter between groups.

### 3.2. Anatomical Outcomes

Treatment with the IV DEX implant resulted in a significant anatomical improvement, with a marked reduction in RME across all groups. For the overall cohort, the mean reduction in the vertical diameter of the largest cyst was −203.3 ± 176.1 µm (*p* < 0.001). When stratified by CEA classification, a statistically significant reduction was observed in group 1 (−227.3 µm; *p* < 0.001), group 2 (−224.8 µm; *p* < 0.001), and group 3 (−135.2 µm; *p* = 0.002). No statistically significant differences in cyst reduction were detected among groups (*p* = 0.100). A moderate correlation was found between baseline cyst diameter and magnitude of reduction following treatment (r = −0.49; *p* < 0.001), indicating greater anatomical response in eyes with larger baseline cysts. Among the cohort, 11 patients (22.0%) developed ocular hypertension following IV DEX. In all these subjects, return within normal intraocular pressure limits was achieved with medical therapy.

### 3.3. Functional Outcomes

**Figure 2:**
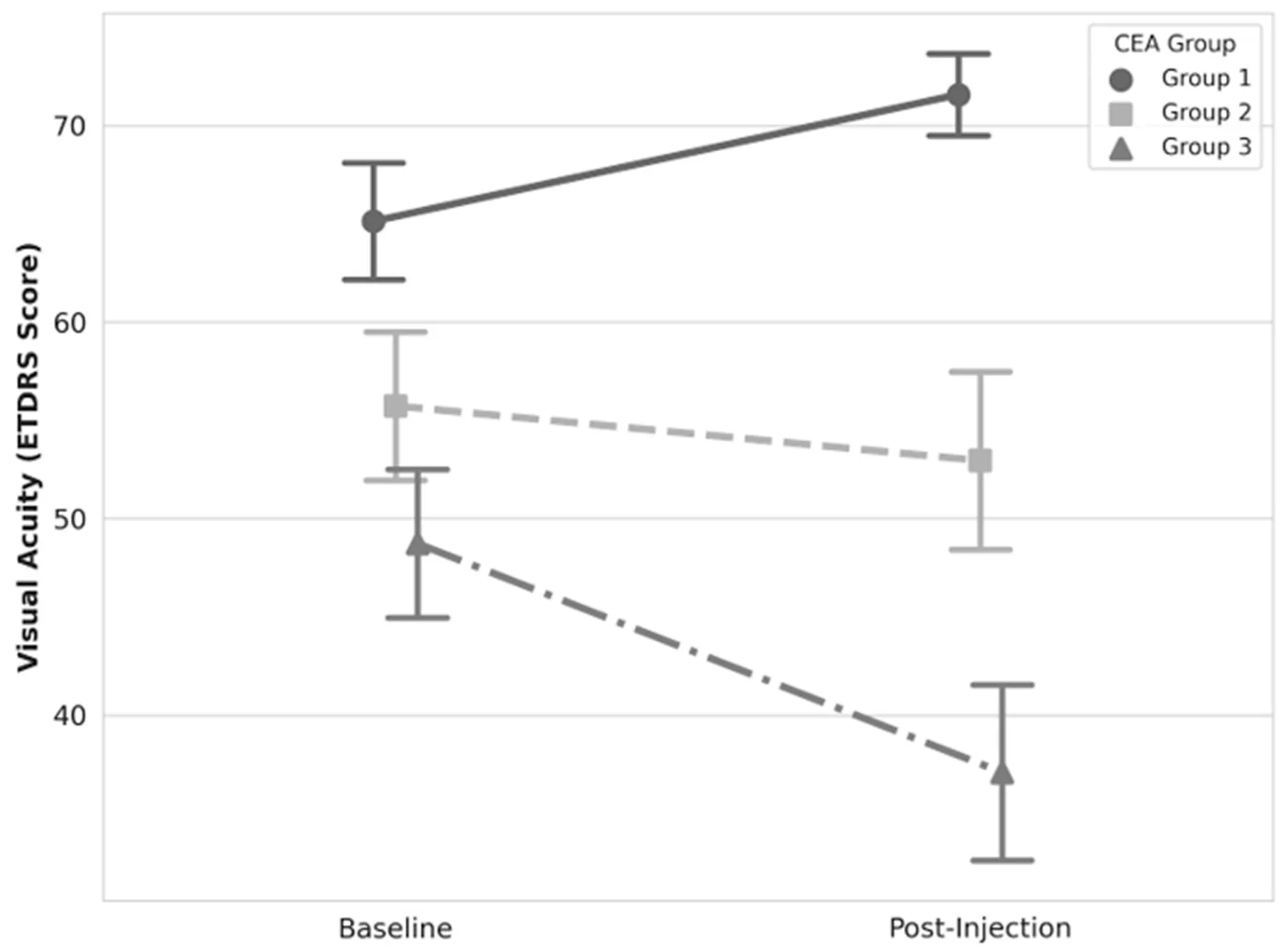
Visual acuity changes by CEA groups (ETDRS score).

Baseline BCVA was significantly higher in group 1 compared with group 3 (*p* = 0.001), whereas no significant differences were observed when comparing either group with group 2 (group 1 vs. group 2: *p* = 0.060; group 3 vs. group 2: *p* = 0.220). In the overall cohort, mean BCVA change was −1.8 ± 16.0 ETDRS letters (*p* = 0.286), indicating no significant functional variation after treatment. However, stratification by CEA classification revealed markedly different visual outcomes (Figure 2).

Functional outcome after IV DEX was strongly influenced by baseline outer retinal integrity. Eyes with preserved outer retinal anatomy (group 1) were the only subgroup demonstrating significant functional improvement, with a mean gain of +6.4 ± 11.1 ETDRS letters (*p* = 0.002). In contrast, eyes with isolated EZ disruption (group 2) showed functional stability, with a non-significant mean change of −2.8 ± 12.4 letters (*p* = 0.183). Eyes with combined EZ disruption and RPE atrophy (group 3) experienced the poorest outcomes, exhibiting a significant mean loss of −11.7 ± 20.9 ETDRS letters (*p* = 0.014). Functional outcomes according to the CEA classification are detailed in Table 3.

**Figure 3:**
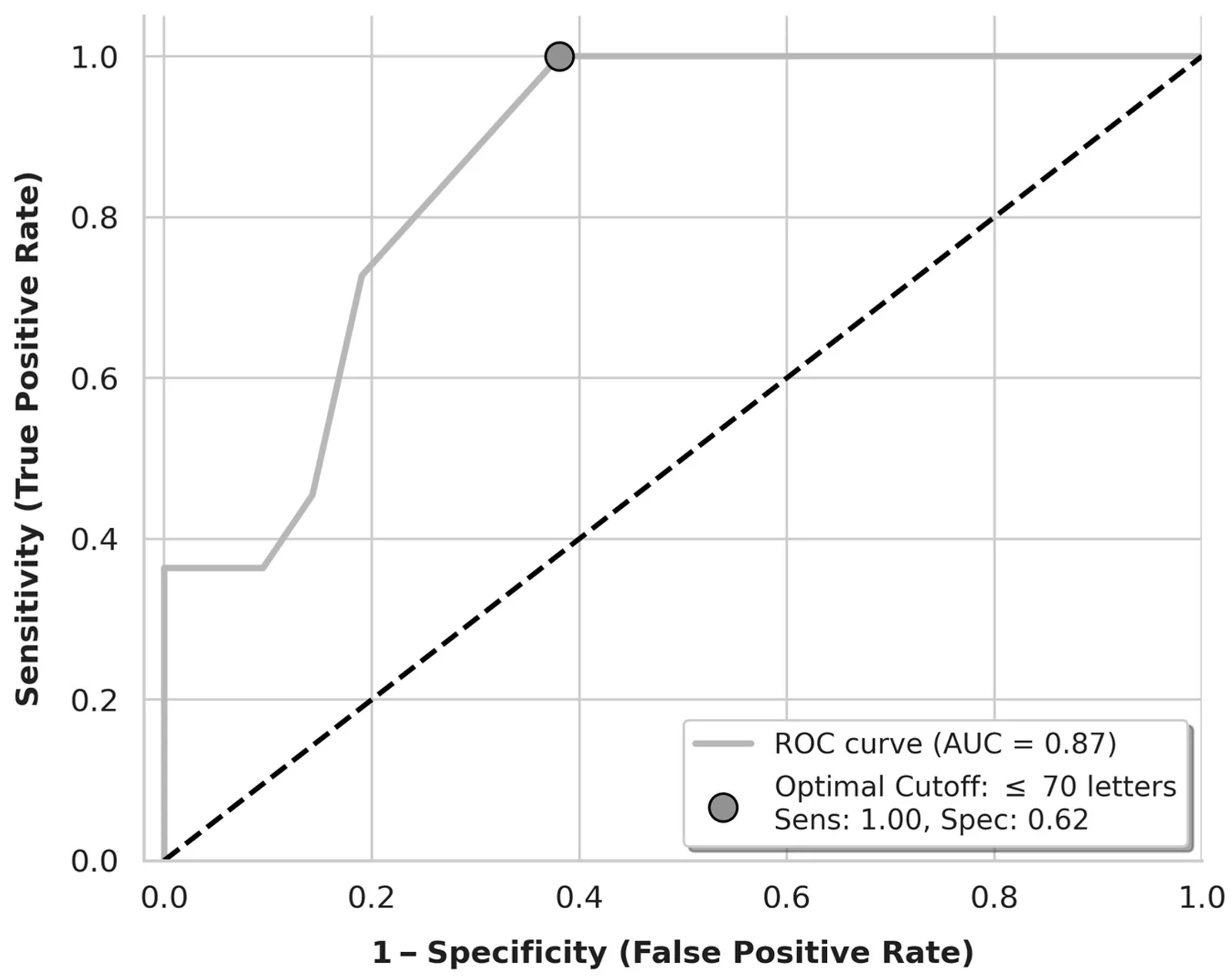
Receiving Operating Characteristics (ROC) curve analysis for prediction of visual improvement (≥5 letters) in group 1 (intact outer retina). The dashed diagonal black line represents the line of no discrimination (random chance).

When evaluating clinical improvement (defined as a gain of ≥5 ETDRS letters) among the various groups, the values were respectively 34.4% in group 1, 14.5% in group 2 and 0.0% in group 3. Given the favorable prognosis of group 1, a subgroup analysis was performed to identify predictors of visual improvement. ROC curve analysis demonstrated excellent predictive accuracy (AUC = 0.87) for baseline BCVA (Figure 3). The optimal prognostic cutoff was identified at 70 ETDRS letters (approximately 20/40 Snellen equivalent; sensitivity 100%; specificity 62%). Treatments in group 1 were stratified by this threshold: eyes below 70 ETDRS letters at baseline demonstrated a mean change of +10.2 ± 11.5 letters, while eyes above it resulted in a mean change of +1.0 ± 4.2 letters (*p* = 0.018).

### 3.4. Multivariable Analysis

Multivariable linear mixed-effects modeling was performed to assess the independent prognostic value of CEA parameters on functional outcome after adjusting for baseline BCVA, as reported in Table 4. Baseline cyst diameter was not a significant predictor of visual outcome. Conversely, EZ disruption emerged as a significant negative predictor. Furthermore, RPE atrophy exerted an additional detrimental effect on functional prognosis (Table 4).

## 4. Discussion

### 4.1. Anatomical Improvement

The primary therapeutic endpoint in RM has historically been the resolution of RME, based on the assumption that RME improvement translates into functional recovery [17]. However, long-term landmark studies, including the 10-year anti-VEGF review by Finger et al. [13] and the triamcinolone study by Shields et al. [14], have consistently shown that despite substantial RME improvement, visual acuity often progressively declines over time. In this context, our study provides validation of the CEA classification as a prognostic tool for functional outcomes following IV DEX therapy in patients with RM [16]. Our results confirm the well-established anatomical efficacy of the DEX implant in reducing RME, in line with previous reports [11,18]. However, functional improvement was strictly dependent on the integrity of the outer retinal layers.

### 4.2. Functional-Anatomical Dissociation

We observed a clear functional–anatomical dissociation in eyes with advanced microstructural damage, in which marked morphological improvement failed to translate into visual gain. This observation is further supported by the absence of an association between baseline cyst parameters and functional outcome in the multivariable model, where cyst diameter was not a significant predictor (*p* = 0.226). These findings challenge the traditional reliance on central subfield thickness (CST) as a surrogate endpoint in RM clinical studies and a commonly reported secondary outcome in major studies [19,20]. Our data suggest that intraretinal fluid volume is only relevant to visual prognosis in the presence of structural integrity of the outer retina. The dissociation between edema resolution and visual outcome observed in our cohort can be interpreted within the pathophysiological framework recently proposed by our group [10]. While RM has long been regarded primarily as a radiation-induced vasculopathy, accumulating evidence supports the concept of a concomitant direct insult to the neurosensory retina, resulting in glial dysfunction and photoreceptor loss. Our findings provide clinical validation of this model: IV DEX effectively targets the vascular-inflammatory component of RM, restoring blood–retina barrier function and reducing edema, but is unable to reverse advanced neurodegenerative damage once EZ disruption has occurred. This paradigm explains the divergent functional outcomes observed across the three CEA groups.

### 4.3. Preserved Outer Retina and Ceiling Effect

Eyes with preserved outer retinal integrity (group 1) demonstrated significant visual improvement (+6.4 ETDRS letters), despite having the best baseline BCVA. While this group exhibited the highest overall visual gain, we identified a baseline BCVA of 70 ETDRS letters as an exploratory pivotal prognostic cutpoint. The subgroup analysis revealed that the recovery is strictly dependent on baseline BCVA, consistent with the “ceiling effect” commonly described in landmark diabetic macular edema trials, in which poorer baseline vision is typically associated with greater functional gain following edema resolution [21,22]. This result underscores that prompt intervention—before EZ disruption—may represent the most critical determinant of functional recovery, but it also relates to the baseline BCVA of each patient.

### 4.4. Structural Biomarkers of Visual Prognosis

In contrast, eyes with isolated EZ disruption (group 2) exhibited functional stability (−2.8 letters) despite significant anatomical improvement. Although visual gain was not achieved, stabilization remains clinically meaningful when compared with the rapid and progressive visual deterioration that characterizes untreated RM [23,24]. Nevertheless, the absence of functional improvement confirms that EZ disruption represents a prognostic threshold beyond which visual recovery becomes unlikely. The substantial reduction in RME observed in this group further emphasizes that visual potential is ultimately driven by photoreceptor health rather than retinal thickness [25,26]. Within this framework, EZ integrity emerges as a key biomarker defining the therapeutic window of opportunity, distinguishing treatments that yield visual stabilization from those capable of achieving true recovery. Most critically, eyes with combined EZ disruption and RPE atrophy (group 3) experienced significant visual loss (−11.7 letters) despite RME improvement. In multivariable analysis, RPE atrophy exerted the strongest negative prognostic effect, with a significant additive impact on visual outcome (Table 4). Given the central role of the RPE in supporting photoreceptor metabolism, its atrophy reflects a fundamental breakdown of the photoreceptor/RPE physiology and marks profound, advanced tissue damage [27].

In this scenario, the lack of functional prognostic value of the C component raises an important question regarding its role within the classification. Nevertheless, we believe that its inclusion remains clinically relevant for capturing the dual clinical presentation of RM. Retaining the C component serves as a practical reminder for clinicians: it emphasizes that the integrity of the outer retina is a major determinant of the functional therapeutic window. Recognizing this biological boundary is essential for establishing realistic clinical expectations and guiding patient care. Consequently, these findings raise important considerations regarding sustained aggressive intravitreal therapy. In the presence of combined EZ disruption and RPE atrophy, continued treatment may warrant careful reconsideration of the expected functional benefit of continued intensive intravitreal therapy, particularly in relation to treatment burden and potential adverse effects.

### 4.5. Limitations of the Study

The main limitations of this study include its retrospective design, variability in the number of injections per patient, and heterogeneity in injection intervals. Nevertheless, the use of linear mixed-effects modeling allowed us to account for repeated measures and intra-patient correlation, strengthening the robustness of the observed associations. Furthermore, the absence of an untreated control group precludes our ability to quantify the true magnitude of vision loss that dexamethasone therapy may have prevented. Additionally, while tumor-specific parameters were recorded, precise dosimetric data regarding the exact radiation dose delivered specifically to the macula and optic disc were not evaluated. Another possible limitation of this study is that our population received exclusively IV-DEX as a standard treatment of RM, whereas most centers treat this disease using primarily anti-VEGF agents. The choice of treating these patients with IV-DEX is driven by the emerging pathophysiological concept of radiation-induced neuroinflammation. In addition, the proposed baseline BCVA cutoff of 70 ETDRS letters was derived from a limited subgroup within our cohort. Consequently, independent prospective validation of this cutoff is required.

## 5. Conclusions

The CEA classification represents a reproducible, comprehensive and reliable OCT-based tool for the stratification of RM, especially in the evaluation of overall functional outcomes after IV DEX. EZ disruption reflects advanced photoreceptor damage and is an independent negative predictive factor of visual recovery. Moreover, the presence of RPE atrophy predicts a visual decline despite anatomical improvement. In conclusion, integration of the CEA classification into routine clinical practice may enhance prognostic stratification and support informed clinical decision-making, treatment planning, and realistic functional expectations. Furthermore, the prognostic insights provided by the proposed structural biomarkers lay the necessary groundwork for the future development of comprehensive, stage-based therapeutic algorithms for the management of RM.

## Figures and Tables

**Table 1:** Tumor and treatment characteristics in the study population.

Feature	Mean ± SD
Tumor height (mm)	5.1 ± 2.3
Tumor largest basal diameter (mm)	13.6 ± 4.3
Tumor location	
Superior	12 (24%)
Nasal	12 (24%)
Inferior	13 (26%)
Temporal	13 (26%)
Distance from the fovea (mm)	7.8 ± 1.4
Dose at tumor apex (Gy)	85 ± 5.3
Dose at tumor base (Gy)	378 ± 36.5
Delivery time (days)	4.7 ± 1.3

SD: standard deviation.

**Table 2:** Distribution of intravitreal dexamethasone injection events and unique patients.

**Distribution of Injections per Patient**	**Number of Patients**
1 injection	26
2 injections	15
3 injections	5
4 injections	1
5 injections	1
6 injections	2
**Unique patients per CEA group**	
Group 1	12
Group 2	13
Group 3	13

**Table 3:** BCVA values at baseline and 4 months (±2 weeks) after IV DEX in the three groups according to the CEA classification.

Groups	Pre-Injection BCVA (ETDRS Score)	Post-Injection BCVA (ETDRS Score)	∆BCVA (ETDRS Score)	*p*-Value
Group 1 (intact)				
Mean ± SD	65.1 ± 16.8	71.6 ± 11.8	+6.4 ± 11.1	0.002
Range	20–85	35–85		
Group 2 (EZ alone)				
Mean ± SD	55.7 ± 23.0	52.9 ± 27.5	−2.8 ± 12.4	0.183
Range	0–83	0–85		
Group 3 (combined EZ and RPE)				
Mean ± SD	48.7 ± 18.1	37.1 ± 21.4	−11.7 ± 20.9	0.014
Range	0–74	0–70		

BCVA: best corrected visual acuity; ETDRS: Early Treatment Diabetic Retinopathy Study; EZ: Ellipsoid zone; SD: standard deviation; RPE: retinal pigment epithelium.

**Table 4:** Multivariable linear mixed-effects model assessing predictors of functional outcome following IV DEX.

Predictor Variable	Coefficient (β)	*p*-Value
Baseline cyst diameter	0.01	0.226
EZ disruption	−11.5	0.003
RPE atrophy	−10.4	0.007

EZ: Ellipsoid zone; RPE: retinal pigment epithelium.

## Data Availability

The data presented in this study are available in the article. Eventual additional data are available on request from the corresponding author.

## Abbreviations

The following abbreviations are used in this manuscript:

ART	Automated real time
BCVA	Best-corrected visual acuity
CST	Central subfield thickness
ETDRS	Early Treatment Diabetic Retinopathy Study
EZ	Ellipsoid zone
IV DEX	Intravitreal dexamethasone
OCT	Optical coherence tomography
RM	Radiation maculopathy
RME	Radiation-related macular edema
RPE	Retinal pigment epithelium
UM	Uveal melanoma
VEGF	Vascular endothelial growth factor
